# By integrating single-cell RNA-seq and bulk RNA-seq in sphingolipid metabolism, CACYBP was identified as a potential therapeutic target in lung adenocarcinoma

**DOI:** 10.3389/fimmu.2023.1115272

**Published:** 2023-01-27

**Authors:** Pengpeng Zhang, Shengbin Pei, Zeitian Gong, Yanlong Feng, Xiao Zhang, Fang Yang, Wei Wang

**Affiliations:** ^1^ Department of Thoracic Surgery, The First Affiliated Hospital of Nanjing Medical University, Nanjing, China; ^2^ Department of Breast Surgery, The First Affiliated Hospital of Nanjing Medical University, Nanjing, China; ^3^ Department of Ophthalmology, Charité – Universitätsmedizin Berlin, Campus Virchow-Klinikum, Berlin, Germany

**Keywords:** lung adenocarcinoma, sphingolipid, CACYBP, tumor immune microenvironment, immunotherapy

## Abstract

**Background:**

Lung adenocarcinoma (LUAD) is a heterogeneous disease with a dismal prognosis for advanced tumors. Immune-associated cells in the microenvironment substantially impact LUAD formation and progression, which has gained increased attention in recent decades. Sphingolipids have a profound impact on tumor formation and immune infiltration. However, few researchers have focused on the utilization of sphingolipid variables in the prediction of LUAD prognosis. The goal of this work was to identify the major sphingolipid-related genes (SRGs) in LUAD and develop a valid prognostic model based on SRGs.

**Methods:**

The most significant genes for sphingolipid metabolism (SM) were identified using the AUCell and WGCNA algorithms in conjunction with single-cell and bulk RNA-seq. LASSO and COX regression analysis was used to develop risk models, and patients were divided into high-and low-risk categories. External nine provided cohorts evaluated the correctness of the models. Differences in immune infiltration, mutation landscape, pathway enrichment, immune checkpoint expression, and immunotherapy were also further investigated in distinct subgroups. Finally, cell function assay was used to verify the role of CACYBP in LUAD cells.

**Results:**

A total of 334 genes were selected as being most linked with SM activity for further investigation, and a risk model consisting of 11 genes was established using lasso and cox regression. According to the median risk value, patients were split into high- and low-risk groups, and the high-risk group had a worse prognosis. The low-risk group had more immune cell infiltration and higher expression of immune checkpoints, which illustrated that the low-risk group was more likely to benefit from immunotherapy. It was verified that CACYBP could increase the ability of LUAD cells to proliferate, invade, and migrate.

**Conclusion:**

The eleven-gene signature identified in this research may help physicians create individualized care plans for LUAD patients. CACYBP may be a new therapeutic target for patients with advanced LUAD.

## 1 Introduction

According to the GLOBOCAN 2020 study, lung cancer is the largest cause of cancer-related mortality globally, accounting for 11.4% of new cancer cases and 18% of cancer deaths (1). Non-small-cell lung cancer (NSCLC) is the most prevalent kind of lung cancer, accounting for around 85% of all occurrences (2). Meanwhile, the most common histological subtype of NSCLC is LUAD. LUAD is characterized by significant heterogeneity and a challenging tumor microenvironment (TME) (3). Conventional pathology stages do not totally predict a patient’s prognosis for NSCLC. Therefore, the development of novel and reliable prognostic models may help in assessing the risk of LUAD patients and offering customized immunotherapy and chemotherapy regimens.

Sphingolipids are physiologically active lipids that are abundant in eukaryotic cells and keep cell membrane fluidity and barrier function in tact (4). Ceramide (Cer), sphingosine -1-phosphate (S1P), sphingosine (Sph), sphingomyelin, and glycosphingolipids are only a few of the sphingolipids that are often present in living things and play crucial structural roles in cell membranes. Sphingolipids are bioactive lipids that may participate in signal transduction for a number of critical physiological functions (5, 6). Several biological activities, including cancer cell proliferation, migration, and invasion, depend on members of the sphingolipid family (7). An in-depth study has been done on sphingolipids and their derivatives as possible therapeutic targets in the fight against cancer. Cer, Sph, and S1P are the three primary important sphingolipid compounds. S1P mostly encourages cell survival, whereas Cer and Sph primarily induce cell cycle arrest and promote cell death. The “sphingolipid-rheostat” controlling the balance between pro-apoptotic Cer/Sph and pro-survival S1P, has been proposed as a novel approach to treating tumors (8, 9). Dihydroceramide also builds up in cells by preventing ceramide desaturation. This phenomenon involves controlling autophagy, particularly autophagy-induced cancer cell death (10). Changes in sphingolipid production may influence a number of signaling pathways, encouraging or preventing the growth of tumors (11–14). A more precise categorization of patients will result from more research and knowledge of the sphingolipids-associated genes, which will also assist in better monitoring overall survival rates and enhancing medication response.

Individualized treatment was made feasible by single-cell RNA-sequencing (scRNA-seq), a potent approach for investigating the mechanisms behind malignancy heterogeneity and development (15, 16). Thus, we employed scRNA-seq and bulk RNA-seq to detect the SRGs signature in LUAD, and based on the median risk scores, we separated the patients into high- and low-risk categories. There was a significant difference in prognosis between the two groups, and nine GEO datasets were chosen to confirm the accuracy of our research. We also looked at the signature’s predictive power for immunotherapy, drug sensitivity, tumor mutational burden (TMB), and the immune microenvironment. Finally, we reduced CACYBP expression *in vitro* to examine its effect on LUAD cell migration and proliferation. Our results provide fresh insight into how SM affects LUAD and open the door to more accurate patient classification and identification, which aid in the development of prognostic biomarkers and novel molecular targets for LUAD gene therapy.

## 2 Materials and methods

### 2.1 Acquisition of raw data

The scRNA-seq data for LUAD, containing 12 LUAD samples, were downloaded from GEO database (GSE150938). The training cohort consisted of LUAD RNA expression patterns, gene mutations, and associated clinical data that were obtained from the TCGA database (n=516). For use as validation sets, the GEO expression profiles of the following genes were simultaneously downloaded: GSE13213, GSE26939, GSE29016, GSE30219, GSE31210, GSE37745, GSE42127, GSE68465, and GSE72019. All of the expression data were converted to TPM format for easier dataset comparison. After that, batch effects were removed using the “sva” package’. Prior to analysis, all data were converted using Log2. SRGs were found in the GeneCards database (https://www.genecards.org/ ), and a total of 122 SRGs with relevance scores greater than 10 were selected for further investigation.

### 2.2 scRNA-seq data processing and cell annotation

Using “seurat” R tools, we checked the scRNA-seq data for accuracy. Genes expressed in at least three single cells, cells with between 200 and 7,000 genes, and cells with more than 10% mitochondrial genes were filtered out to preserve high-quality scRNA-seq data. For further investigation, 46286 suitable cells in total were chosen. Using a linear regression model and the “Log-normalization” technique, the remaining cells were scaled and normalized. Following data normalization, the top 3,000 hypervariable genes were discovered using the “FindVariableFeatures” tool. We used the “FindlntegrationAnchors” function of the canonical correlation analysis (CCA) approach to remove the batch effects that would have interfered with downstream analysis since these data were derived from several samples. In order to appropriately integrate and scale the data, we then utilized the “IntegrateData” and “ScaleData” functions. Principal component analysis (PCA) dimensionality reduction was used to find the anchor points. The t-distributed stochastic neighbor embedding (t-SNE) algorithm was used to test the top 20 PCs in order to find the meaningful clusters. We obtained 20 cell clusters after using the “FindNeighbors” and “FindClusters” functions (Resolution =0.8) and then displayed these clusters as a “t-SNE” diagram. Based on the cell cycle markers included in the “seurat” package, cell cycle heterogeneity along the clusters was assessed. Cell cycle scores were calculated using the “CellCycleScoring” tool based on the expression of G2/M and S-phase markers. The “FindAIIMarkers” tool of the “Seurat” program was used to determine the differentially expressed genes (DEGs) of each cluster. We utilized the cutoff threshold values and modified *P<*0.01 and log2 (foldchange) >0.25 criterion to determine which genes served as markers for each cluster. Based on each cluster’s canonical marker genes, cell types were carefully confirmed after annotation in accordance with earlier findings (17, 18).

### 2.3 AUCell

The “AUCell” R package, which analyzed the active status of gene sets in scRNA-data, was used to assign SM activity scores to each cell lineage. Using the “ggplot2 R” software to visualize, the cells were then divided into high- and low-Sphingolipid-AUC groups based on the median values of the AUC score. Utilizing the “GSVA” package, Gene Set Variation Analysis (GSVA) was carried out to investigate the heterogeneity of diverse biological processes. In order to identify the biological pathways that are enriched in both the high-and low-Sphingolipid-AUC groups, the GSVA was carried out. The bar chart showed all of the noteworthy paths.

### 2.4 ssGSEA and WGCNA analysis

ssGSEA was used to determine the absolute enrichment percentage of a specified gene set in each sample (19, 20). In this study, we employed ssGSEA to assign SM enrichment values to each individual in the TCGA-LUAD. WGCNA is a biological technique for constructing the gene co-expression network of TCGA-LUAD (21, 22), and the “WGCNA” package in R implements this method. The detailed processes were as follows: missing value genes were eliminated using the “goodSamplesGenes” function, the tumor samples were grouped, outliers were eliminated, and a cut line of 120 was defined. After that, a visual determination of the ideal soft threshold for adjacency computation was made. To identify the network’s genetic connectedness, the expression matrix was converted into an adjacency matrix and then into a topological overlap matrix (TOM). On the basis of the variations in TOM, average linkage hierarchical clustering was then carried out. In order to integrate modules with high correlation coefficients (R > 0.25), the hierarchical clustering tree was dynamically pruned to find similar modules. Module eigengenes (MEs) were the principal building block of gene modules, being capable of standing in for all genes in a given module. Pearson correlation was used to determine the relationship between eigengene values and clinical characteristics. Finally, module genes with the most remarkable correlation to sphingolipid score were selected for further analysis.

### 2.5 Construction and validation of the risk scoring

As mentioned above, the TCGA-LUAD series as a pilot analysis, whereas nine GEO datasets served as the validation set. To begin, we took the intersection of the differential genes with the target genes in WGCNA as candidate predictors. We applied univariate analysis on the intersection gene to identify genes that statistically correlate with patient’s OS (*P*< 0.01). Next, LASSO and multivariate regression analysis were performed to further filter for genes and risk coefficients strongly associated with prognosis (23). Each LUAD patient was given a risk score based on the coefficients identified by the multivariate analysis. Based on the median risk score, patients from TCGA-LUAD were divided into high- and low-risk categories. Meanwhile, Kaplan-Meier method was employed to plot survival curves for prognostic purposes, and log-rank tests were performed to determine the statistical significance. Receiver operating characteristic (ROC) curves were used to examine the prediction model’s efficacy; an AUC value of >0.65 indicated excellent performance. The predictive ability of the signature was validated in independent nine GEO datasets utilizing survival analysis and AUC. We employed the “FactoMineR” package to perform PCA dimensionality reduction analysis and visualized the discrepancy between the two risk groups. Cohorts for validation were subjected to a similar procedure.

### 2.6 Assessment of the prognostic model and analysis of mutations

To calculate the probabilities of OS at 1, 3, and 5 years, we developed a nomogram combining the risk score, age, and pathological stage as independent prognostic factors. ROC curves were drawn to evaluate the accuracy of the nomogram. To assess the nomogram’s accuracy, ROC curves were created. Using concordance index analysis, we further assessed the net benefit of the nomogram and clinical characteristics alone. Stratified analysis was used to evaluate the prognostic value of risk score clinical features (age, gender, clinical stage, pathological T and N stage). The TCGA database was used to retrieve gene mutation profiles of LUAD patients, which were then computed using the “maftools” software. The risk score was combined with the comprehensive gene mutation information.

### 2.7 Enrichment analysis

The GSVA made use of the hallmark gene sets “h.all.v7.5.1.symbols.gmt” from MSigDB (https://www.gseamsigdb.org/gsea/msigdb/index.jsp ). Then, the activity of each gene set in each sample was assessed using the GSEABase package (24). The link between model genes and 51 immune genes (25) was then further examined, and the results were shown using a circular heat map. From Mariathasan’s research characteristics, a set of genes positively linked with anti-PD-L1 medication response was gathered (26–28). The GSVA method was used to construct enrichment scores for gene features that were positively related to immunotherapy and the cancer immune cycles, and *P* ***<*** 0.05 were regarded as significantly different between the two groupings. For the examination of connections between the two aforementioned genetic characteristics and risk scores, the R package “ggcor” was utilized.

### 2.8 Tumor immunity and immunotherapy

Based on the expression profiles, the R package “estimate” was used to infer malignant tumor tissues’ stromal and immune cell abundance and tumor purity (29). A higher score indicates a greater percentage of TME components. The present study aimed to see whether or not there were significant differences in survival or responsiveness to treatment between different groups. Machine learning can accurately assess and quantify immunogenicity. The Cancer Immunome Atlas (TCIA) database was used to retrieve the Immunophenoscores (IPS) for LUAD (30). To forecast immunotherapy sensitivity, the IPS was examined. Additionally, immune checkpoints are made up of several molecules that are expressed on immune cells and regulate the level of immune activation. They are very important for limiting excessive immune activation. We compared the levels of expression in both groups of well-known immune checkpoint genes (ICGs). To evaluate the possible response to immunotherapy, the Tumor Immune Dysfunction and Exclusion (TIDE, http://tide.dfci.harvard.edu/ ) algorithm was used (31). We next determined the degree of immune infiltration for LUAD patients in the TCGA database from the TIMER 2.0 database, which contains the results of 7 evaluation methods. These data were applied to quantify the relative fractions of immune cell infiltration in the TME in the form of heatmaps. There are six recognized immune subtypes: wound healing, inflammatory, lymphocyte deficient, immunologically quiet, and TGF-dominant (25). The “ImmuneSubtypeClassifier” software was used to assess six immune subtypes in LUAD samples and compare them to the developed risk model. The differences were examined using the Chi-square test.

### 2.9 The role of CACYBP in LUAD

The expression of CACYBP in pan-cancer was explored using the timer database. In order to investigate variations in survival, patients were split into two groups based on CACYBP expression: high- and low-expression, and finally the correlation between gene expression and enrichment scores of the Hallmark gene sets was explored.

### 2.10 Tissue collection and cell lines culture

The tissue samples collected from the First Affiliated Hospital of Nanjing Medical University were approved by the Medical Ethics Committee (2019-SR-156) and were kept at -80°C. Ten pairs of samples, including tumor tissue (T) and precancerous tissue (N), were collected from LUAD patients undergoing tumor resection. BEAS-2B cells (normal human lung epithelial) and A549 and H1299 cells (human LUAD cell lines) were acquired from the Cell Resource Center of Shanghai Life Sciences Institute. These cells were grown in F12K or RPMI-1640 (Gibco BRL, USA) with 10% fetal bovine serum (FBS), 1% streptomycin, and penicillin (Gibco, Invitrogen, Waltham, MA, USA). 5% CO2, 95% humidity, and 37°C were used to cultivate the cells.

### 2.11 Cell transfection

CACYBP knockdown was generated using small interfering RNAs (siRNAs). In addition, CACYBP siRNA sequences were listed in **Supplementary Table S1**. Briefly, cells were seeded at 50% confluence in a 6-well plate and infected with negative control (NC), and knockdown (siCACYBP). All transfections were carried out with Lipofectamine 3000 (Invitrogen, USA).

### 2.12 Extraction of RNA and real-time PCR

Total RNA from cell lines and tissues was extracted by the manufacturer’s instructions using TRIzol (15596018, Thermo). After that, cDNA was created using the PrimeScriptTMRT kit (R232-01, Vazyme). SYBR Green Master Mix (Q111-02, Vazyme) was used to perform the Real-time polymerase chain reaction (RT-PCR), and the expression levels of each mRNA were normalized to the level of mRNA GAPDH. The 2^−ΔΔCt^ method was used to count the expression levels. Tsingke Biotech (Beijing, China) provided all primers, and **Supplementary Table S1** has full primer sequences.

### 2.13 Colony formation

We transfected 1×10^3^ cells into each well of a 6-well plate and kept the cells alive for 14 days. Before Crystal violet (Solarbio, China) staining, the cells were washed twice with PBS and fixed for 15 minutes in 4% paraformaldehyde.

### 2.14 Wound-healing assay

In 6-well plates, transfected cells were plated and cultured in a cell incubator until they were 95% confluent. In each cultured well, a single straight line was scraped using a sterile 20-L plastic pipette tip, and unattached cells and debris were gently washed away twice with PBS. Finally, we used the Image J software to measure the width of the scratches after taking photos of the scratch wounds at 0h as well as 48h.

### 2.15 Transwell assay

Cell invasion and migration studies were performed *via* transwell assay. The top chamber of 24-wells was filled with treated A549 and H1299 cells (2×10^5^), which were then incubated for 48 hours. To assess the cells’ ability to invade and migrate, the top section of the plate was either precoated with matrigel solution (BD Biosciences, USA) or left untreated. The remaining cells on the bottom layer were then fixed with 4% paraformaldehyde and stained with 0.1% crystal violet after the cells on the top surface were removed (Solarbio, China).

### 2.16 Statistical analysis

R (version 4.1.3) was used to process all of our data and statistics. Utilizing the programs Graphpad and Image J (version 1.8.0), experimental data were processed (version 9.4.0). To compare the survival rates of the two groups, Kaplan-Meier curves with a log-rank test were employed. All survival curves were produced using the R program survminer. Cox regression analysis, both univariate and multivariate, was used to evaluate prognostic variables. Lasso regression was used to identify factors that had a bigger influence on results. We used the R software “ggplot2” to show the data and the R package survival to compute the OS and risk scores. Pheatmap was used to create the heatmap. A two-tailed t-test or a one-way ANOVA was used to identify significant quantitative differences for normally distributed variables. A Wilcoxon test or a Kruskal-Wallis test was used to evaluate if there were significant differences for nonnormally distributed data. Using R software, every statistical analysis was carried out. A statistically significant value is *P*< 0.05.

## 3 Results

### 3.1 The scRNA profiling of LUAD

**Figure 1:**
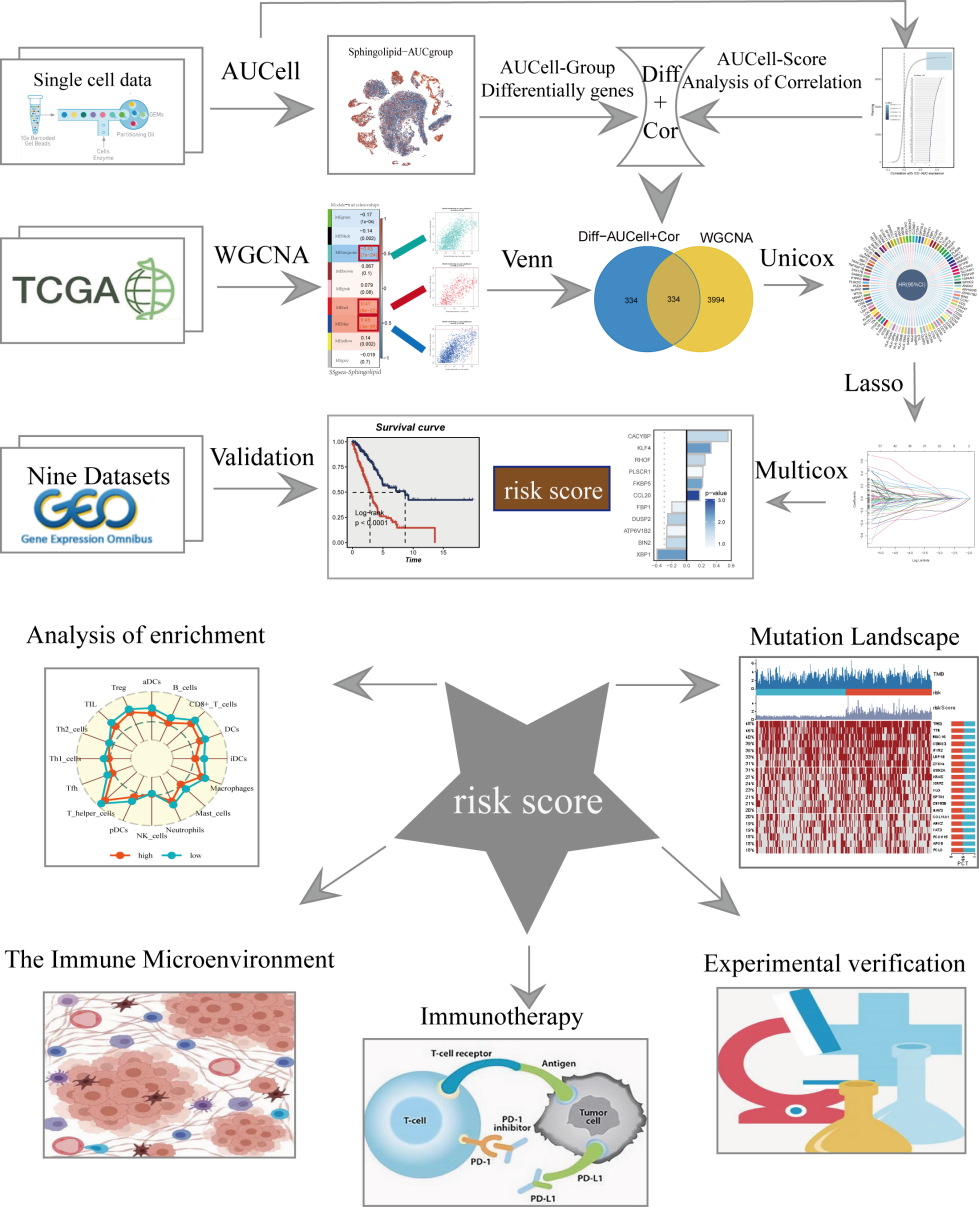
The flowchart of this study.

**Figure 2:**
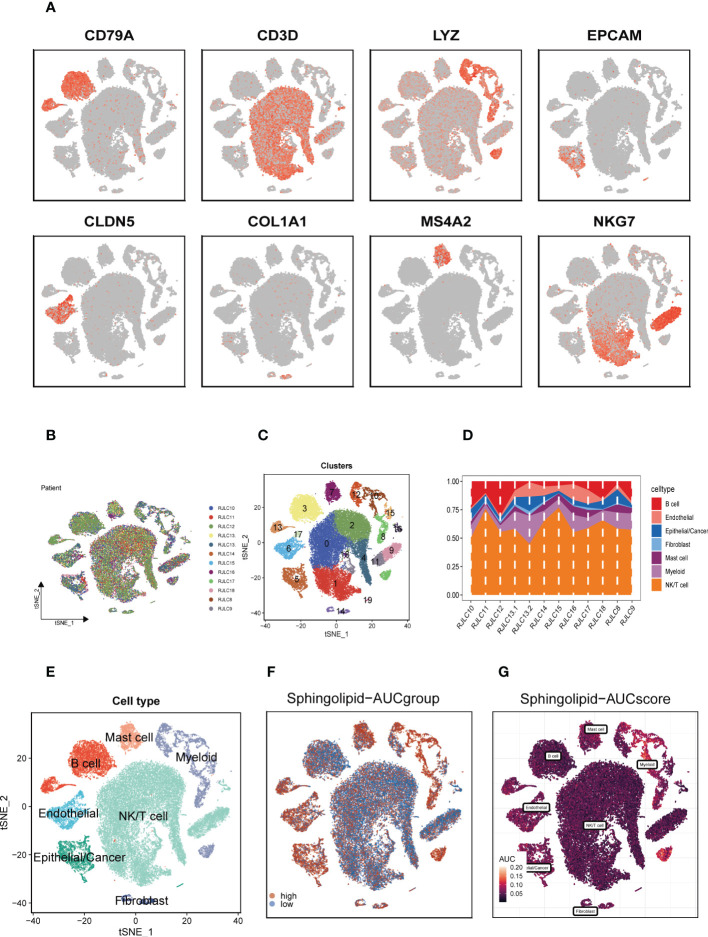
Annotation of single-cell data and SM activity. **(A)** Typical marker genes for each cell group. **(B)** The t-SNE plot showed that all the cells in the 12 LUAD samples. **(C)** The t-SNE plot showed that all the cells in 20 clusters. **(D)** Different proportions of cells in 12 samples. **(E)** The t-SNE map indicates that LUAD samples can be annotated as 7 cell types in the TME (different colors represent different cell types). **(F, G)** AUCell score and groups of SM activity in each cell.

**Figure 1** displayed the study’s flowchart. After a quality check based on the percentage of cell signatures and the expression of the mitochondrial and ribosomal genes, a total of 46286 high-quality cells were deemed suitable for further study. Each sample’s expression characteristics are shown in **Supplementary Figure S1A**. There are statistically significant positive correlations between sequencing depth and total intracellular sequences (R=0.94, **Supplementary Figure S1B**). No discernible variations in cell cycles were seen in the PCA reduction plot **(** **Supplementary Figure S1C** **)**. **Figure 2A** illustrated typical markers for different cell types. There were 12 samples included in the study, and the cell distribution within each sample was generally consistent, indicating that there was no discernible batch impact among samples, which could be used for further investigation **(** **Figure 2B** **)**. Subsequently, the dimensionality reduction methods, namely t-SNE, classified all cell to 20 clusters **(** **Figure 2C** **)**. **Figure 2D** showed the proportion of each cell in the 12 LUAD samples with line plots, among which NK/T cells accounted for the highest proportion. **Figure 2E** illustrated the distribution of each cell population with a t-SNE plot. A total of seven cell types can be found, such as Endothelial cells, Fibroblasts, Myeloid, and tumor cells. Using the “AUCell” R package, the SRGs activity of each cell was determined **(** **Figure 2F** **)**. Higher AUC values were seen in cells that expressed more genes, and these cells were mostly orange-hued myeloid cells **(** **Figure 2G** **)**. All cells were assigned an AUC score for the corresponding SRGs and divided into high- and low-Sphingolipid-AUC groups by AUC score median values.

### 3.2 Identification of the most relevant genes for SM activity and the construction of a risk model

**Figure 3:**
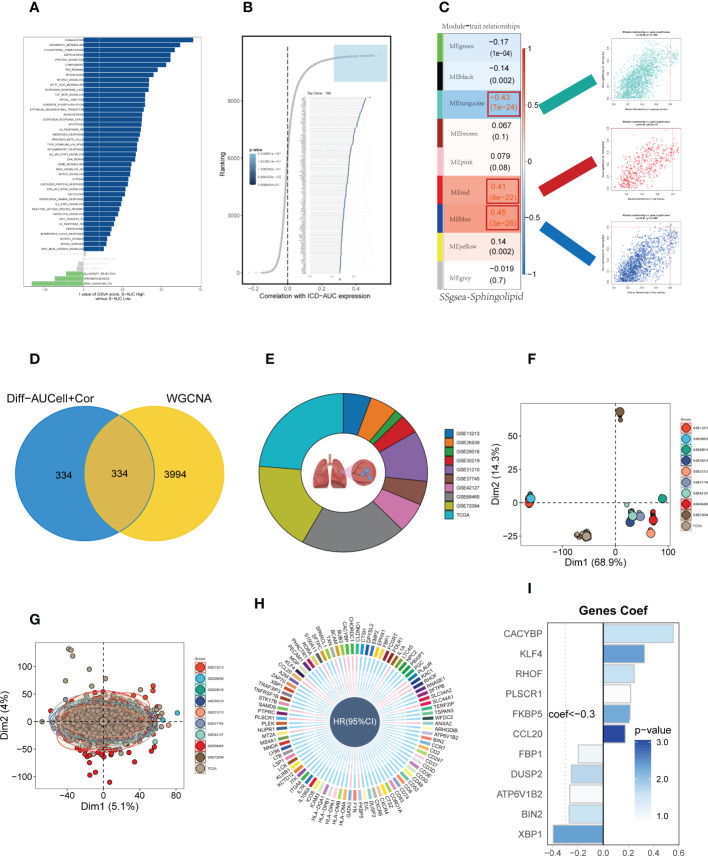
Construction and validation of SM-related prognostic model. **(A)** GSVA showed the enrichment of hallmark gene sets in different SM AUCell groups. **(B)** Correlation analysis between SM-AUCell score and genes. **(C)** WGCNA analysis searched for the modules most associated with SM activity. **(D)** Venn plots identified the genes most associated with SM activity. **(E** The sources of samples and the proportion of sample size in 10 datasets were analyzed. **(F, G)** PCA plots before and after removal of batch effects for 10 datasets. **(H)** Genes significantly associated with prognosis after univariate regression. **(I)** Model genes and coefficients identified by lasso regression and multivariate analysis.


To elucidate the potential biological mechanisms of distinct AUC scores, the GSVA was conducted and results showed that the top five pathways enriched in high Sphingolipid-AUC group were Coagulation, Xenobiotic metabolism, Cholesterol homestasis, Adipogenesis, and Protein secretion **(** **Figure 3A** **)**. We next performed differential analyses to identify DEGs related to SM between high-and low-Sphingolipid-AUC groups and a total of 613 DEGs were selected for the subsequent study. In addition to this we performed correlation analysis to explore the genes most associated with SM metabolic activity **(** **Figure 3B** **)**, the top 150 most associated genes were used for subsequent analysis. In the single-cell analysis, the DEGs and the genes obtained from the correlation analysis were merged to obtain the genes that most affected SM activity (764 in total). Each sample in TCGA-LUAD had its SM score determined using ssGSEA, and WGCNA was used to further search for gene sets that were covarying with SM score. **Supplementary Figure S1D** showed that mean connectivity tends to be stable and the data is more compatible with the power-law distribution when the soft domain value is 7. This makes the data appropriate for further investigation. Nine non-gray modules **(** **Figure 3C** **)** were produced when the minimum number of modules was set to 100, deepSplit to 3, and merging the modules with similarity lower than 0.25 (**Supplementary Figure S1F**). The relationship between each module’s expression and clinical characteristics was assessed. Finally, the 761 genes most associated with SM activity obtained in the single-cell analysis were intersected with the three modular genes most associated with SM activity obtained in WGCNA, and a total of 334 overlapped genes were selected to be analyzed in the following step **(** **Figure 3D** **)**. For better data consistency, we removed the batch effect from the GEO-obtained data and with the TCGA data, **Figure 3E** showed the distribution ratio of the ten data sets, and **Figures 3F, G** showed the PCA plots before and after the batch effect was removed, respectively. After that, TCGA was divided into training and validation sets according to 6:4, and univariate COX analysis was performed, and the results were shown by a circular plot **(** **Figure 3H** **)**, and finally, the risk model consisting of 11 genes was obtained by multivariate COX and lasso regression analysis **(** **Figure 3I**, **Supplementary Figure S1F** **)**. The formula is as follows:

$$risk\;score=\sum_{n=i}^{k}\left(Coef_{i}\exp i\right)$$

Coefi and Expi represented the coefficient and expression of each model gene, respectively, and the risk score for each sample was calculated by the above formula.

### 3.3 Survival analysis and model evaluation

**Figure 4:**
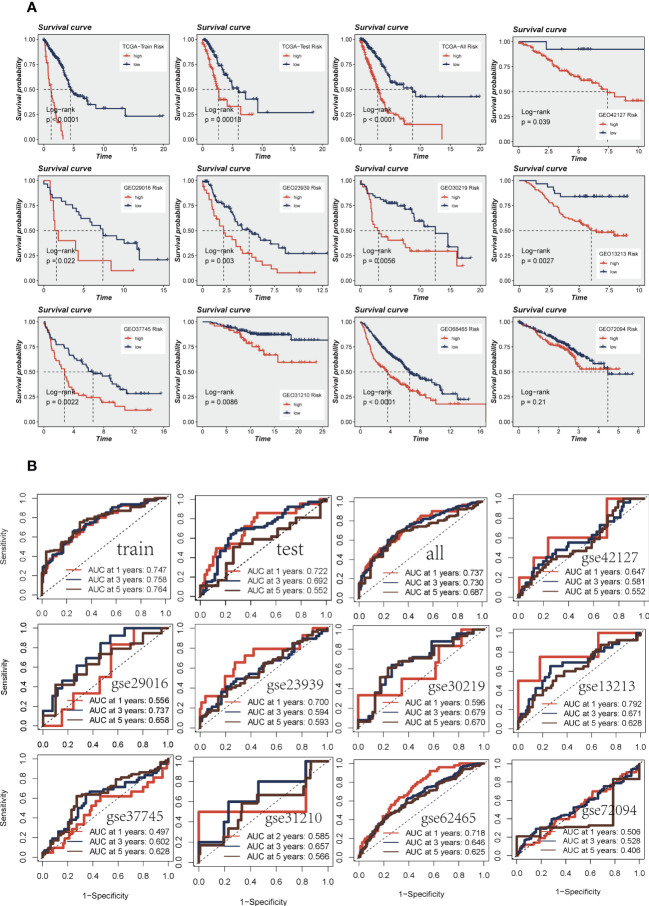
Assessment of risk models. **(A)** Kaplan-Meier prognostic analysis of signatures in the training, testing, whole TCGA, and nine GEO datasets. **(B)** The ROC curve was used to evaluate the performance of the model in the training, testing, whole TCGA, and nine GEO datasets.

Patients were divided into high-and low-risk groups based on median risk values and survival analysis showed a significant difference in survival in the TCGA dataset LUAD patients. In the geo datasets, there were eight datasets with a significant difference in survival (*P<* 0.05, **Figure 4A**). This indicates the reproducibility and stability of the model. The ROC curves showed high AUC values in TCGA, and most of the datasets in the GEO datasets had AUC values greater than 0.6 **(** **Figure 4B** **)**. Ten datasets from TCGA and GEO were subjected to PCA analysis according to the model gene expression, and the final results are presented in **Supplementary Figure S2A-K**. PCA found that According to the risk scores, patients could be distinguished well in two dimensions, which suggested that the model had a promising capacity to stratify risk subtypes in both the TCGA cohort and the GEO cohorts.

### 3.4 Construction and validation of prognostic nomogram integrating risk score and clinicopathological parameters

**Figure 5:**
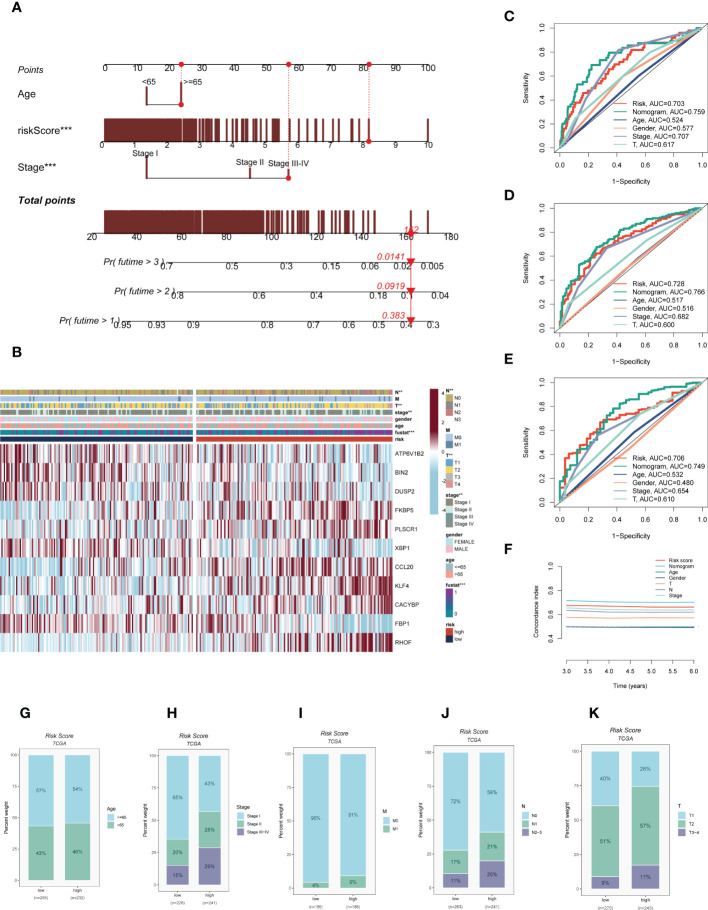
Building a more accurate nomogram. **(A)** Nomogram was constructed by combining clinical features with risk score. **(B)** Heat map incorporating clinical data, model genes. **(C–E)** ROC curves for 1, 3, and 5 years showed AUC values for various clinical factors, risk scores, and nomogram scores. **(F)** The C-index curves were used to evaluate the predictive performance of different clinical characteristics, nomogram scores and risk scores. **(G–K)** The proportion of multiple clinical characteristics in different risk subgroups. :**P < 0.01, ***P < 0.001.

On the basis of the TCGA-LUAD dataset, a predictive nomogram incorporating risk score and clinicopathological characteristics (age and clinical stage) was created to more accurately predict the prognosis of LUAD **(** **Figure 5A** **)**. Clinical outcome parameters were used based on survival at 1, 3, and 5 years. The AUC of the ROC curve for predicting patient prognosis was maintained at around 0.750, indicating that the nomogram’s predictive value was greater than that of any one clinical trait **(** **Figures 5C–E** **)**. C-index curves clarified that clinical features combined with the risk score could predict the prognosis with more sensitivity compared to a single indicator **(** **Figure 5F** **)**. The above results determined that the nomogram is suitable for clinically predicting the prognosis of LUAD. A heatmap of correlations between prognostic signatures of risk score and clinicopathological outcomes was also generated. According to the heatmap, the risk score presented correlated positively with clinical status, clinical stage, T stage, and N stage (*P*< 0.05, **Figure 5B**), while no statistical difference was found between other clinical features such as age and clinical stage. The distribution of various clinical traits in various groups was then compared and shown as a percentage bar plot. We also assessed thepredictive performance of risk categories in LUAD patients stratified by various clinical characteristics. We saw that patients in the high-risk category consistently had considerably inferior outcomes across a wide range of clinical categories, which suggested the universal applicability of the prognostic model **(** **Figures 5G–K** **)**.

### 3.5 Mutational landscape

**Figure 6:**
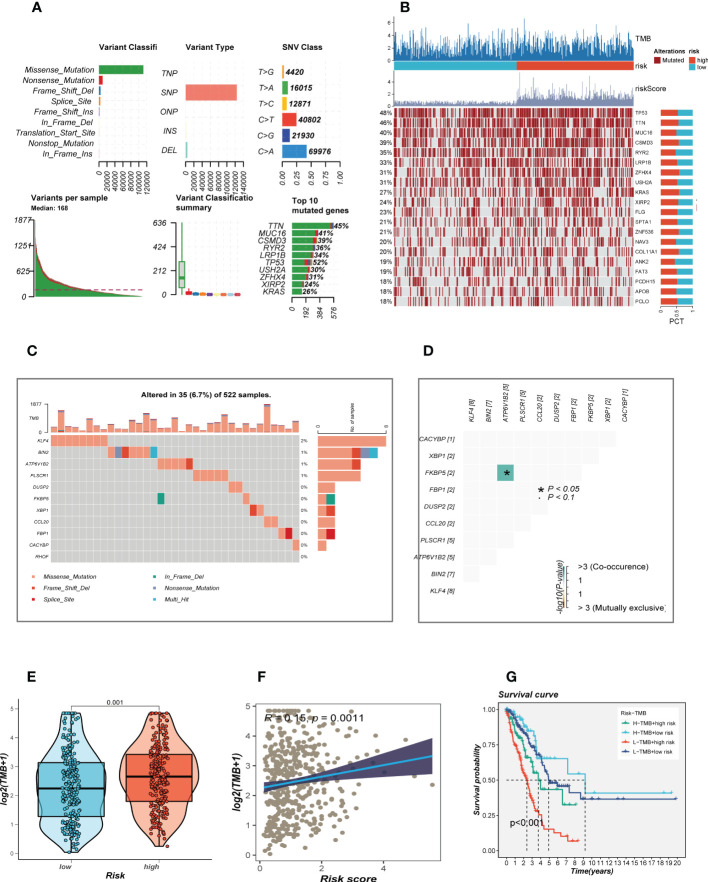
Landscape of LUAD sample mutation profiles. **(A)** Description of the statistical measurement mutation details, among which the most common mutation type was a missense mutation. SNP occupied an absolute proportion compared with insertion or deletion, and C>A occurred more frequently than in other classifications of forms. The horizontal histogram listed the top 10 mutated genes in melanoma. **(B)** Mutation landscape of the top 20 genes with mutation frequency in differential risk subgroups. **(C)** Mutation landscape of model genes. **(D)** Co-mutation or co-exclusion relationships among model genes. **(E)** Comparison of tumor mutation burden (TMB) between different risk groups. **(F)** Correlation analysis between risk score and TMB. **(E)** Survival differences for four different subgroups (H-TMB+high-risk, H-TMB+low-risk, L-TMB+high-risk, and L-TMB+low-risk).

Given that genetic mutation also played a key part in cancer patients’ tailored treatment. We examined the somatic mutation profiles of risk. As shown in **Figure 6A**, the top 3 most frequent mutant genes were TTN, MUC16, and CSMD3. The distributions of gene mutations among risk groupings were then further examined. **Figure 6B** showed that genes with the top 20 high-frequency mutations have a higher mutation frequency in the high-risk group, such as TP53, TTN, and CSMD3. We next looked at the distribution of mutations in model genes in TCGA-LUAD and showed that model genes were mutated in a total of 6.7% of samples, with KLF4 having the highest mutation frequency, which may be a key factor affecting the prognosis of LUAD patients **(** **Figures 6C** **)**. furthermore, co-mutations of model genes were examined and the results showed that KFBP5 and ATP6V1B2 were co-mutated (*P<* 0.05, **Figures 6D**). **Figure 6E** showed that there is a significant difference in the TMB of patients in the high- and low-risk groups, with higher TMB in the high-risk group. Next, we classified patients into four categories based on median TMB values and median risk values (H-TMB+high-risk, H-TMB+low-risk, L-TMB+high-risk, and L-TMB+low-risk), and the results show that patients with low-risk and high mutations had the best prognosis (**Figure 6F**).

### 3.6 Pathway enrichment analysis

**Figure 7:**
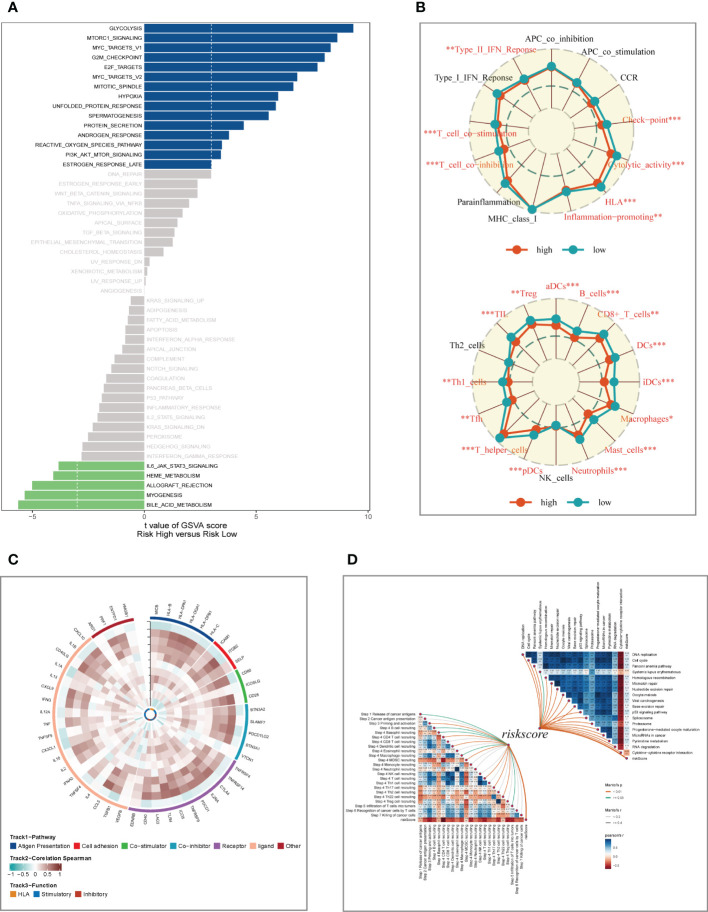
Enrichment analysis and functional annotation. **(A)** GSVA shows the enrichment of hallmark gene sets in different risk subgroups. **(B)** The ssGSEA algorithm was used to evaluate the differences in immune cells and immune-related functions between high- and low- risk subgroups. **(C)** Heat map of correlation between model genes and immune genes. **(D)** Correlation of risk scores with ICB response signature and each step of the tumor-immune cycle. *P < 0.05, **P < 0.01, ***P < 0.001.

To explore the underlying mechanism that could lead LUAD patients in the high-risk group to a poor prognosis. Analysis of hallmark pathway gene signatures highlighted that the high-and low-risk groups showed some differences. A direct comparison of Risk-High versus Risk-Low revealed the top 5 enriched signatures in the high-risk group included Glycolysis, mTORC1 signaling, C-MYC target, G2M checkpoint, and E2F targets **(** **Figure 7A** **)**. Glycolysis is an essential condition for the occurrence and development of tumors (32). High-risk samples may present a worse prognosis for LUAD patients by upregulating the glycolytic pathway. MTORC1 is an effective pathway to promote tumor progression, and targeting MTORC1 may become a therapeutic target for LUAD patients in the high-risk group (33). Furthermore, c-Myc is necessary for tumorigenesis (34), almost often, Myc may increase transcription (35), which showed that LUAD cells could be susceptible to Myc inhibition. Extremely crucial nuclear transcription factors involved in controlling the cell cycle are encoded by the E2F family (36, 37). According to clinical research, E2F family members are directly linked to the incidence, growth, proliferation, and apoptosis of cancerous tumors such as gastric, pulmonary, liver, esophagus, prostate, bladder, and ovarian cancer (36, 38). In order to control cell proliferation, the G2M checkpoint also functions as a cell cycle regulatory route. High G2M checkpoint pathway activation has been linked in studies to considerably worse survival in people with pancreatic cancer (39). As a result, these pathways, which were more prevalent in the high-risk group, may play a crucial role in controlling tumor development in LUAD. To explore the TME of high-and low-risk group samples,we used ssGSEA to evaluate the composition of immune cells between two risk groups **(** **Figure 7B** **)**. Patients in low-high exhibited considerably more partial innate immunity cells (like DC cells and macrophages) and adaptive immunological population (like B cells and T cells) than patients in other groups. Additionally, LUAD patients in the low-risk group had usually higher enrichment scores of immune-related activities produced. To further investigate the variations in immune response across various risk categories, we performed correlation analysis using model genes and classical immune-related genes **(** **Figure 7C** **)**, and the results showed that high-risk genes (HR>1) such as CACYBP and CCL20 were negatively correlated with immune genes, while low-risk genes such as DUSP2 and FBP1 were negatively correlated with immune genes, which may explain the significant differences in immune microenvironment and survival prognosis between high-and low-risk groups. The biological performance of the chemokine system and other immunomodulators was assessed using the tumor immune cycle as a critical indication (26, 40, 41). As a result, we examined the relationship between the activity of tumor immune steps and various risk groups **(** **Figure 7D** **)**, and we found that there was a strong negative relationship between risk score and the activity of the majority of cycle steps, including cancer cell antigen expression (step 2), initiation and activation (step 3), and immune cell trafficking into tumors (step 4). These immune cell recruitment steps include T cell recruitment, CD4 T cell recruitment, CD8 T cell recruitment, Th1 recruitment, DC cell recruitment, and Th22 (step 7). The relationship between immune checkpoint blockade (ICB) response signatures and SRGs risk scores was then investigated further. Risk scores had a positive correlation with the majority of ICB signals but a negative correlation with the interaction between cytokines and their receptors.

### 3.7 Differences in tumor immune microenvironment in different risk groups

**Figure 8:**
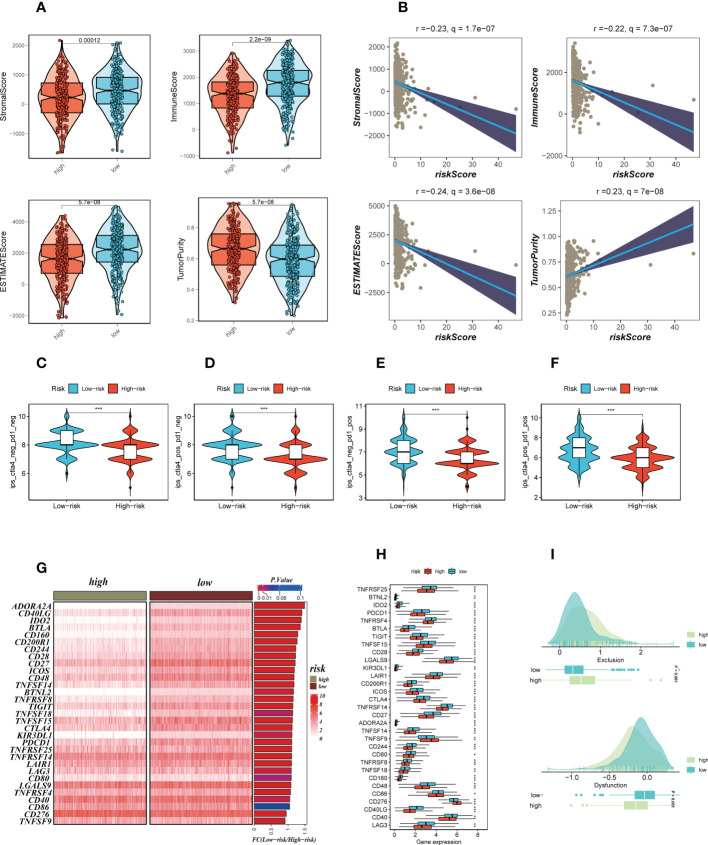
Analysis of TIME and immune checkpoint. **(A)** The violin plot demonstrated the difference in ESTIMATE Score, Immune Score, Stromal Score and tumor purity calculated using the ESTIMATE algorithm between the two risk subgroups. **(B)** The correlations in ESTIMATE Score, Immune Score, Stromal Score and tumor purity calculated using the ESTIMATE algorithm between the two risk subgroups. **(C–F)** The low-risk group has significantly greater IPS, IPS-CTLA4-neg-PD-1-neg, IPS-CTLA4-pos-PD-1-neg, IPS-CTLA4-neg-PD-1-pos, and IPS-CTLA4-pos-PD-1-pos. Note: *P < 0.05, **P < 0.01, ***P < 0.001. **(G, H)** Heat map and box plot showed that differences in immune checkpoint gene expression between high- and low-risk subgroups. **(I)** TIDE between those LUAD patients at high-and low-risk statue.

We calculated parameters using the ESTIMATE technique to assess differences in the TME among different risk groups. According to our research, patients with low-risk had greater stromal, immune, and ESTIMATE scores (stromal+immune) than those with high-risk, although the high-risk group had higher tumor purity **(** **Figure 8A** **)**. The correlation findings indicated a positive relationship between the risk score and the distribution of each TME component **(** **Figure 8B** **)**. Immunotherapeutic effectiveness may change depending on the tumor progression due to the different immune infiltration levels. In light of the aforementioned results, we investigated whether the prognostic model could forecast LUAD patients’ responses to ICIs. Individuals who could benefit from immunotherapy may be found using the IPS. It was expected that tumor samples from these individuals would have a positive immune response to either PD-1/PD-L1 or CTLA4 inhibitors, or both **(** **Figure 8C–F** **)**. The IPS scores of the patients in the low-risk group were much higher, indicating that they would benefit most from this kind of immunotherapy. The findings of our investigation into the variations in ICGs between the high-and low-risk groups were then shown as heat maps **(** **Figure 8G** **)** and boxplots **(** **Figure 8H** **)**, respectively. Most ICGs, including CD40LG, LAG3, PD-1, and others, were more strongly expressed in the low-risk group, while TNFS9 was more significantly expressed in the high-risk group. Evasion of the immune system is one of the important features of cancer that depends on the successful survival of this tumor in the human body. TIDE, a technique to identify variables that underpin tumor immune escape pathways, may function as a useful biomarker for anticipating immunotherapy response in patients with a variety of malignancies, particularly those treated with ICIs. With anti-PD1 and anti-CTLA4 treatments, a greater tumor TIDE prediction score is linked to a poorer ICB response as well as a worse patient survival. According to our research, the low-risk group benefited better after immunotherapy, had a lower Exclusion score, and had a decreased likelihood of tumor immune escape **(** **Figure 8I** **)**. Therefore, it is speculated that patients in the low-risk group are more suitable for immunotherapy.

### 3.8 Reassessment of immune infiltration and analysis of CACYBP

**Figure 9:**
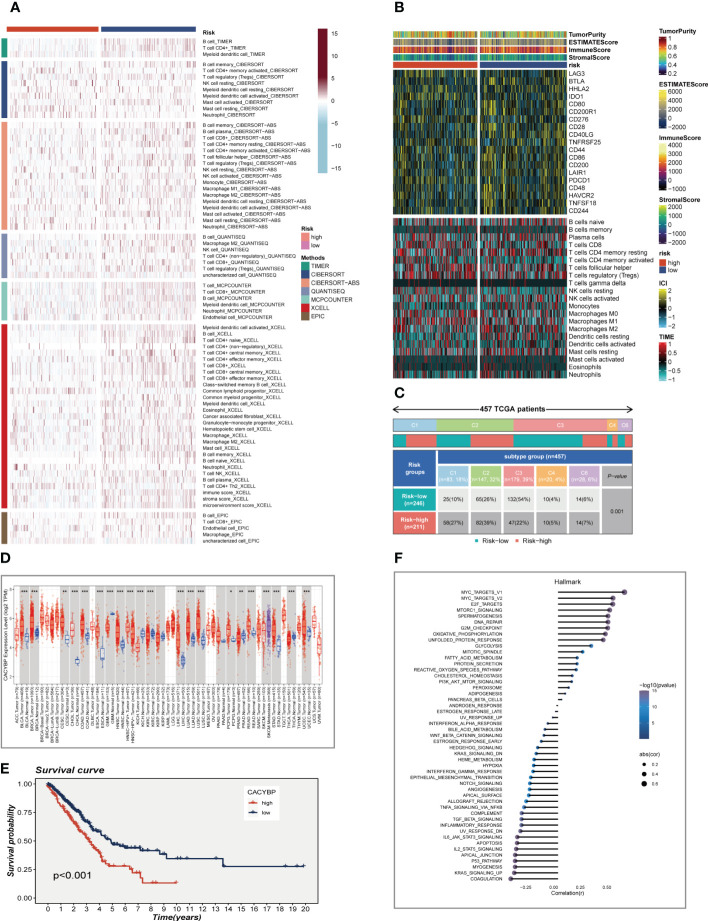
Analysis of immunotherapy and the immune microenvironment. **(A)** Seven algorithms assess differences in immune infiltration status between different risk groups. **(B)** A heat map showed the differences in TME scores, immune checkpoint expression and immune cell infiltration calculated by Cibersort among different risk subgroups. **(C)** Relationship between high- and low-risk groups and six immune subtypes. **(D)** Boxplot showed CACYBP expression in pan-cancer. **(E)** Survival analysis showed the effect of CACYBP expression on prognosis. **(F)** Correlation between CACYBP expression and 50 hallmark pathways. *P < 0.05, **P < 0.01, ***P < 0.001.

In order to further verify the difference in immune cell infiltration in different risk groups, we used seven algorithms to evaluate the immune infiltration of different risk groups. The results are shown in **Figure 9A**. It can be seen that there was higher immune cell infiltration in the low-risk group, such as B cell, T cell, DC cell, etc. This means that the TME of the low-risk group tends to form a hot tumor with a better effect on immunotherapy. **Figure 9B** showed the heat map of TME scores, immune checkpoints, and CIBERSORT calculated immune cell infiltration scores, which also illustrates that there are more highly expressed immune checkpoints and higher levels of immune cell infiltration in the low-risk group. Following that, the association between model grouping as well as immune subtypes was investigated, as demonstrated in **Figure 9C**. Immune subtypes C1, C2, and C6 were more common in the high-risk group. Previous research has revealed that the immunological subtype C3 group had the best prognosis, and C3 was mostly found in the low-risk category, which was consistent with the previous findings. Further research was done on the CACYBP with the highest HR value in the signature. CACYBP was found to be significantly expressed in tumor tissues in the majority of malignancies when we looked at the relative expression of CACYBP in pan-cancerous tumors and nearby tumors using the TIMER database **(** **Figure 9D** **)**. We divided TCGA-LUAD patients into high- and low-CACYBP expression groups and found that patients with high-CACYBP expression had a worse prognosis **(** **Figure 9E** **)**. Similarly, the gse31210 dataset showed similar results **(** **Supplementary Figure S3A** **)**. To explore the reasons for this, we analyzed the relationship between CACYBP expression and the hallmark gene sets **(** **Figure 9F** **)** and found that the top five pathways that positively correlated with CACYBP expression were Myc targets v1, Myc target v2, E2F targets, mTORC1 signaling, and Spermatogenesis, which may be important factors affecting tumor progression. Further experimental verification proved our conclusion.

### 3.9 Experimental validation of CACYBP

**Figure 10:**
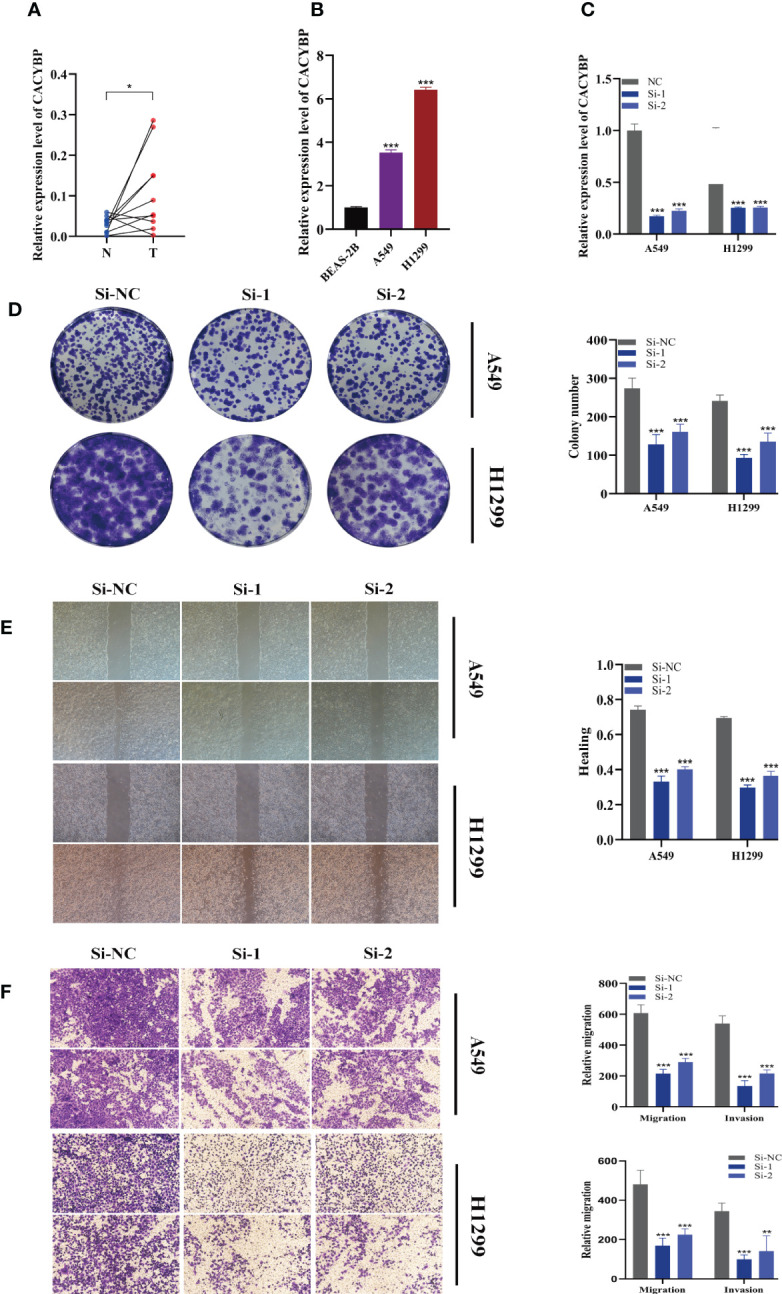
The role of CACYBP in LUAD. **(A)** Relative expression of CACYBP in tumor and paracancerous tissues in LUAD and CACYBP was highly expressed in tumor tissues compared with adjacent tissues **(B)** CACYBP was highly expressed in LUAD cell lines compared to healthy human lung epithelial BEAS-2B cell line. **(C)** RT-qPCR was performed to measure the relative expression of CACYBP in LUAD cells transfected with si-RNAs or negative control (NC). **(D)** Colony formation assay displayed that cell with reduced CACYBP expression exhibited a significant reduction in the numbers of colonies, compared with the NC group. **(E)** Scratch-wound healing assay. A significant reduction in migration rate was observed in cells with reduced CACYBP gene expression. **(F)** Transwell assay showed that downregulation of CACYBP expression inhibited the migration and invasion capacity of LUAD cells. To demonstrate the accuracy and reproducibility of the results, three independent experiments were performed in two LUAD (A549, H1299) cell lines and all data were presented as the means ± SD of three independent experiments. *P < 0.05, **P < 0.01, ***P < 0.001.

The expression of CACYBP was evaluated in ten pairs of LUAD carcinomas and adjacent tissues **(** **Figure 10A** **)**, and it was found that CACYBP was significantly highly expressed in the tumor tissues, which was similarly validated in our cell line experiments **(** **Figure 10B** **)**. And then, we employed the qRT-PCR technique to measure the level of CACYBP expression 5 days after transfection in order to gauge the effectiveness of siRNA knockdown of CACYBP in A549 and H1299 cell lines **(** **Figure 10C** **)**. Clonal formation tests after that showed that CACYBP knockdown significantly decreased the LUAD cell line’s capacity to form colonies **(** **Figure 10D** **)**. These findings suggested that CACYBP knockdown prevented LUAD cell growth. The knockdown of CACYBP dramatically impaired the migration and invasion capacity of LUAD cells, as detected by the wound healing assay **(** **Figure 10E** **)**. In line with the results of the wound healing assay, LUAD cells transfected with si-CACYBP exhibited weaker migratory invasive ability in the transwell assay **(** **Figure 10F** **)**. All of these findings suggested that CACYBP operated in LUAD as a pro-oncogenic regulator.

## 4 Discussion

Immune system plays important role in the development of cancers, as well as immunotherapy (42). Notably, metabolic molecules are greatly influencing the immune environment (43) and thus the progression of the disease (44). Sphingolipids influence cell signal transmission by functioning as secondary messengers and controlling a number of biological processes, and they are crucial for maintaining the fluidity and functionality of membrane barriers (45). Nearly all of the key metabolic enzymes that control the relative abundance of sphingolipids have been found and cloned during the last several years by a large number of researchers. These enzymes’ varying activity alter how cancer progresses, which has an impact on therapy (46). Sphingolipids have been found to be crucial in the development of many diseases, including lung cancer. Immune checkpoints have been discovered and developed, which has raised the possibility of defeating cancers. However, only few patients benefit from tumor immunotherapy, and there are definite (47). Tumor immunotherapy research now focuses on ways to increase immunotherapy’s effectiveness and broaden the population that benefits from treatment. There is mounting evidence that the TME’s heterogeneity is to blame for the variations in treatment results (48). The comprehension of the TME has substantially benefited from the emergence of scRNA-seq technology (17). In the current investigation, we used single-cell sequencing to examine 12 LUAD samples, and seven different cell types were identified in total. The SM gene set, which was downloaded from the GeneCards database, was used to calculate SM activity using the AUCell algorithm, and it was discovered that myeloid cells had the highest levels of SM activity. This suggested that SM may regulate myeloid cells to affect tumorigenesis and development. The most important genes controlling SM activity were then identified. In the TCGA database, we also created a distinct prognostic prediction signature for LUAD patients, and the signature employed in this study had 11 SRGs: ATP6V1B2, BIN2, DUSP2, FKBP5, PLSCR1, XBP1, CCL20, KLF4, CACYBP, FBP1, and RHOF. The unfavorable genes in the signature model were CCL20, FKBP5, PLSCR1, RHOF, KLF4, and CACYBP, while other genes showed protective action on the prognosis of LUAD patients. The survival study of eight external GEO cohorts revealed statistically significant differences, the predictive potential of the signature was confirmed, and the AUC value of the ROC curves indicated that the signature had some predictive power.

A recent study found a clear link between genetic modifications and the formation of neoantigens and immunotherapeutic effects (49). However, the results of this study showed that patients in the low-risk group have less TMB and the high-risk group had more mutations in the high-frequency genes. Next, we split the patients into four groups (H-TMB+high-risk, H-TMB+low-risk, L-TMB+high-risk, and L-TMB+low-risk). The low-risk and high-mutation group had the best prognosis, which may provide fresh guidelines for clinical assessment of patient prognosis. To explore the underlying mechanisms underlying the differences in survival between the different risk groups, a series of pathway enrichment analyses were further explored. According to the GSVA data, the high-risk group had considerably enriched Glycolysis, Myc, and mTORCH pathways, which may have an impact on the incidence and growth of cancers. Further investigation of ssGSEA enrichment analysis revealed that the low-risk group had more immune cell infiltration and immune-related function enrichment. We next used correlation analysis to examine the association between model genes and immune-related genes in order to investigate the probable mechanism of action. The findings revealed a substantial negative correlation between face and factor and the high-risk gene CACYBP, demonstrating that the immune system’s activation was constrained in the high-risk group’s TME. Tumor-fighting immune cells are ineffective. Finally, we explored the correlation between risk score and tumor immune cycle, and ICB response signature. The result demonstrated that when the risk score increased, the immune checkpoint signature was activated and the tumor immune cycle process was suppressed, creating a suppressive immune microenvironment.

The extracellular matrix, cancer-associated fibroblasts, new blood vessels, endothelial cells, and tumor-infiltrating immune cells make up the TME, which may encourage tumor degradation, enhance tumor invasiveness, and heighten the antitherapeutic response (50–52). In order to better understand how TME affects tumor prognosis, we compared immune cell infiltration between high-risk and low-risk LUAD patients. Seven algorithms were used to assess the immune cell infiltration in various risk groups, and the results revealed that the low-risk group’s tumors had a higher level of immune cell infiltration in the surrounding tissue and were more likely to develop hot tumors. This finding also explained why the low-risk immune function was simpler to activate against tumor progression and to support a favorable prognosis. ICGs are now widely acknowledged as being predictive indicators of responsiveness to PD-1/PD-L1 inhibitors (53). TIDE, a recently identified immunotherapy predictor, has shown superior predictive ability compared to other biomarkers or indications (54). We investigated the distinctions between this signature and ICGs in order to demonstrate that this signature may be a biomarker of immunotherapy response. We discovered that patients in the high-risk category for SRGs had considerably higher TIDE scores as well as lower scores for T cell dysfunction and PD-1 protein expression, which indicated that these patients would benefit less from immunotherapy. TCIA analysis was carried out to investigate the effects of PD-1 and CTLA-4 treatment in various risk groups in order to better assess the variations in immunotherapy’s effectiveness in various risk groups. According to the findings, the low-risk group would benefit more from immunotherapy since their IPS score was much higher than that of the high-risk group.

Surprisingly, our research found that of the nine modeled genes, CACYBP had the highest HR value, and additional survival analysis showed that high CACYBP expression was associated with a poor prognosis in LUAD patients. Then, we used the A549 and H1299 LUAD cell lines to examine how well the cells functioned. The results showed that the knockdown of CACYBP in LUAD cell lines significantly decreased cell invasion, migration, and proliferation.

The current research contains a number of drawbacks. First, public datasets were used to build this signature. Large-scale prospective clinical trials are required to confirm the predictive potential. The capacity of the SRGs signature to predict the response to immunotherapy was indirectly assessed in this research since patients who had received immunotherapy were not studied. We only conducted basic cell function assays in our work, thus more animal experiments are required for confirmation. In conclusion, using integrated analysis of single-cell and bulk RNA-sequencing, we created and validated a unique prognostic signature made up of eleven SRGs that has the potential to be a prognostic biomarker and predict patients’ responses to immunotherapy in LUAD. Our findings provide light on the function of SRGs in LUAD patient prognosis and treatment responsiveness.

## Data availability statement

The original contributions presented in the study are included in the article/**Supplementary Material**. Further inquiries can be directed to the corresponding authors.

## Ethics statement

The studies involving human participants were reviewed and approved by Ethics Committee of Jiangsu Province People’s Hospital, the First Affiliated Hospital of Nanjing Medical University. The patients/participants provided their written informed consent to participate in this study.

## Author contributions

PZ, SP, ZG, and FY contributed conception and design of the study; ZG, XZ and FY collected the data; PZ and FY performed the statistical analysis; PZ, SP, and FY wrote the first draft of the manuscript; WW and FY gave the final approval of the version to be submitted. All authors contributed to manuscript and approved the submitted version. 
