# Supplementary material for: By integrating single-cell RNA-seq and bulk RNA-seq in sphingolipid metabolism, CACYBP was identified as a potential therapeutic target in lung adenocarcinoma
Source: Front Immunol. 2023 Jan 27;14:1115272. doi: 10.3389/fimmu.2023.1115272 (PMC9914178; doi:10.3389/fimmu.2023.1115272)
Supplement: Supplementary file 1 [file DataSheet_1.docx]

Supplementary Material

# Supplementary Figures


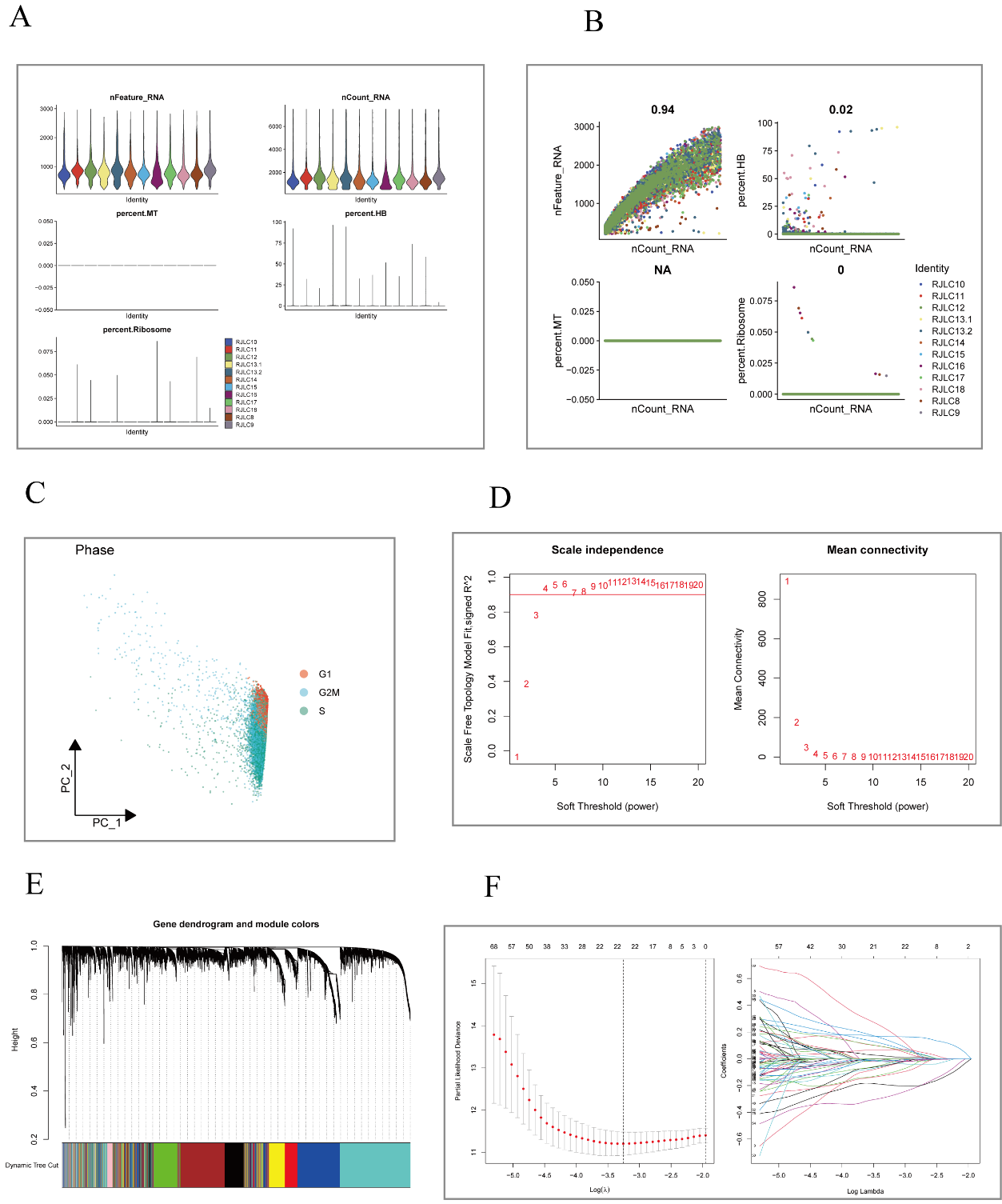


**Supplementary Figure S1.** Quality control of single-cell sequencing data and model construction. (A) The distribution of gene expression levels, sequencing depth, the percentage of red blood cell genes, the percentage of mitochondrial genes and the percentage of ribosome genes in the 12 samples. (B) Correlation between sequencing depth and gene expression levels, the percentage of mitochondrial genes, the percentage of red blood cell genes, the percentage of ribosome genes. (C) Cell cycle related genes were used for dimensionality reduction clustering. (D) The soft domain value is 7, the data more consistent with the power-law distribution, and mean connectivity tends to be stable. (E) The minimum number of modules were set to 100 and deepSplit to 3 and merging the modules with similarity lower than 0.25. (F) Twenty-two genes were selected for multivariate regression analysis using Lasso regression.


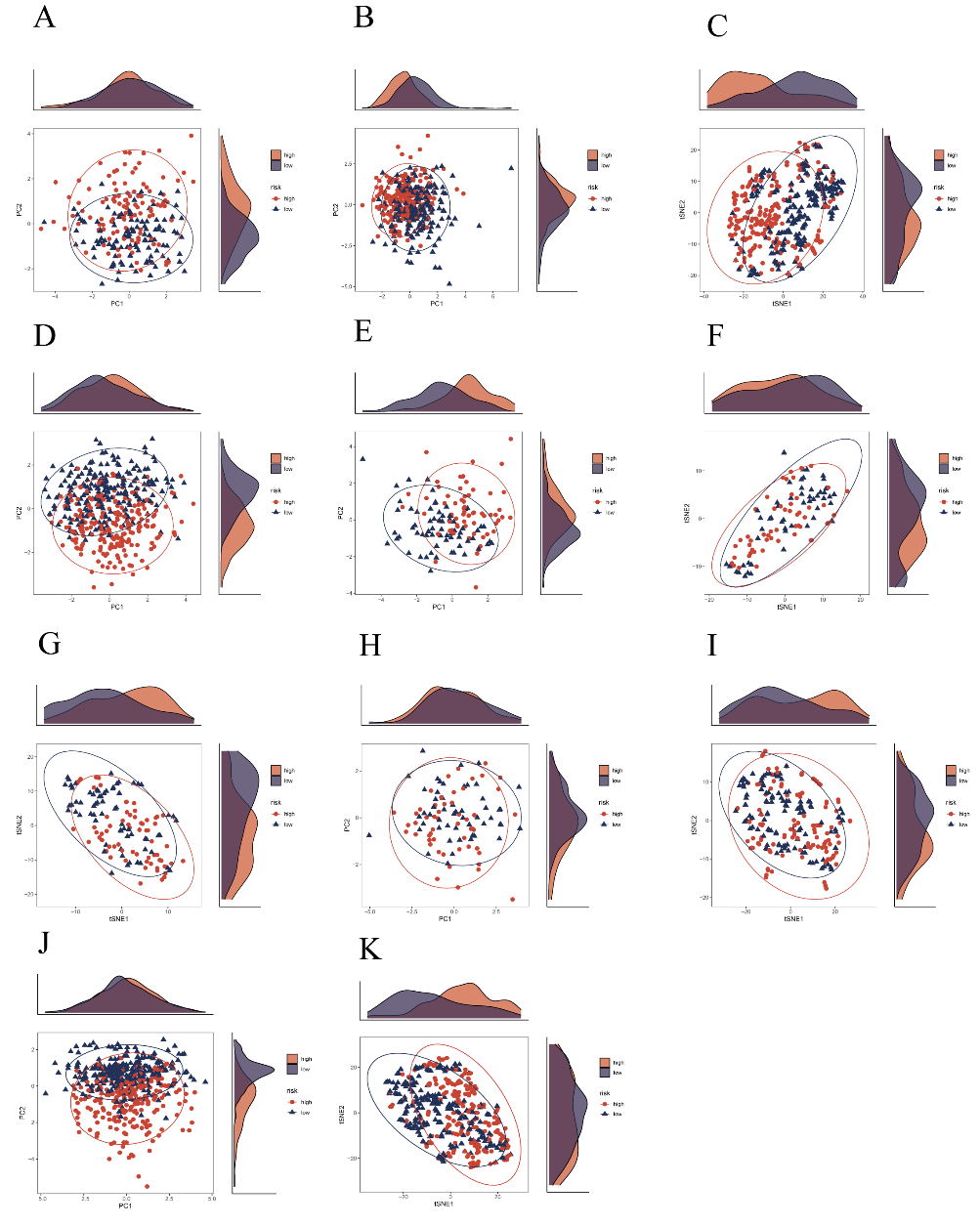


**Supplementary Figure S2.** PCA analysis showed that the distribution of samples in the high-and low-risk groups. (A-K) Ten datasets from TCGA and GEO were subjected to PCA analysis according to the model gene expression.


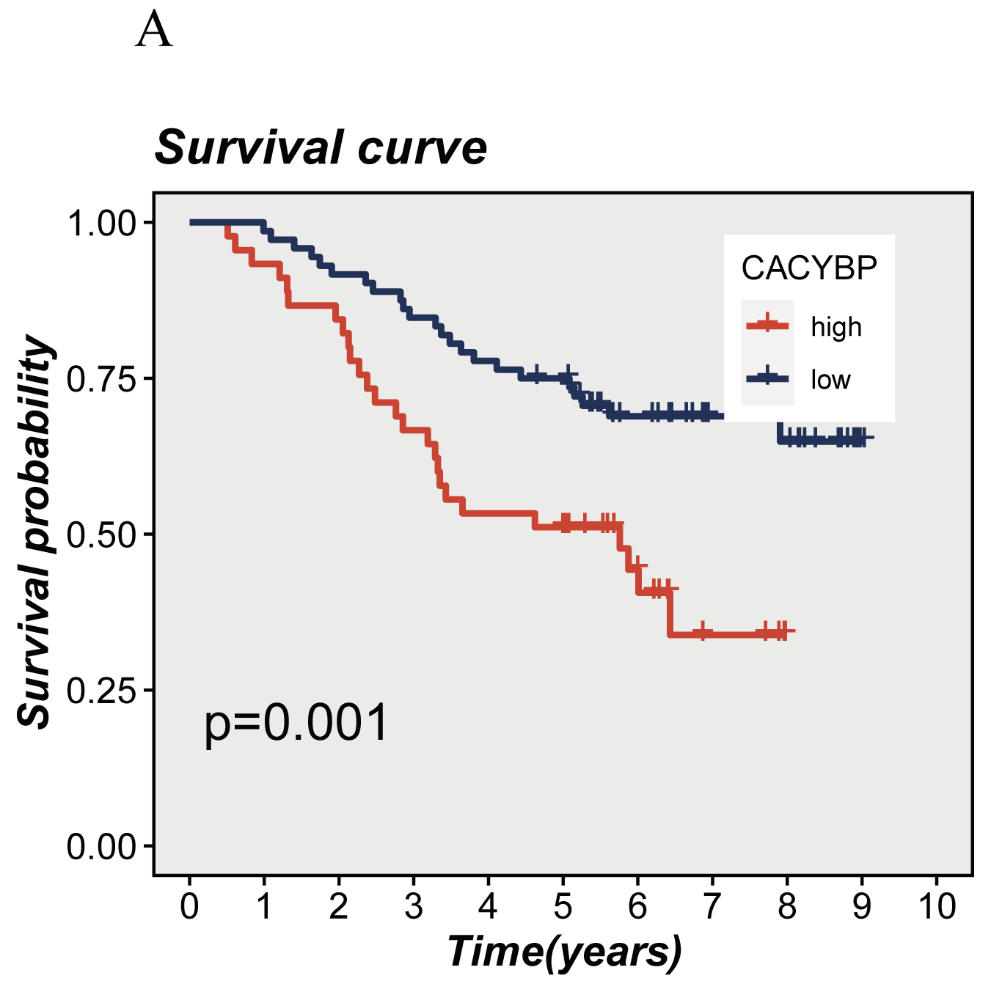


**Supplementary Figure S3.** A survival curve plot. (A) GSE31210 dataset was used to verify the effect of CACYBP on the prognosis of LUAD patients.
