# Supplementary material for: By integrating single-cell RNA-seq and bulk RNA-seq in sphingolipid metabolism, CACYBP was identified as a potential therapeutic target in lung adenocarcinoma
Source: Front Immunol. 2023 Jan 27;14:1115272. doi: 10.3389/fimmu.2023.1115272 (PMC9914178; doi:10.3389/fimmu.2023.1115272)
Supplement: Supplementary file 2 [file Table_1.docx]

| **Oligonucleotides** | **Nucleotide sequence (5'-3')** |
| --- | --- |
| **siRNA** |  |
| Scramble control | GCUUCGCGCCGUAGUCUUA |
| Si-CACYBP-1 | AAGAGTTACTCCATGATTGTG |
| Si-CACYBP-2 | AATCAAGAACAAGATGCAAC |
|  |  |
| **Primer** |  |
| GAPDH | GGCCTCCAAGGAGTAAGACC (forward) |
|  | AGGGGAGATTCAGTGTGGTG (reverse) |
| CACYBP | GGGATCAGTCAGATAAGTT (forward) |
|  | TTCAGTGTCATAGGAGGG (reverse) |
|  |  |

**Table S1. Oligonucleotides used in research**
